# An Overview on How Exercise with Green Tea Consumption Can Prevent the Production of Reactive Oxygen Species and Improve Sports Performance

**DOI:** 10.3390/ijerph19010218

**Published:** 2021-12-25

**Authors:** Hadi Nobari, Saber Saedmocheshi, Linda H. Chung, Katsuhiko Suzuki, Marcos Maynar-Mariño, Jorge Pérez-Gómez

**Affiliations:** 1 HEME Research Group, Faculty of Sport Sciences, University of Extremadura, 10003 Caceres, Spain; jorgepg100@gmail.com; 2 Department of Physical Education and Sports, University of Granada, 18010 Granada, Spain; 3 Department of Physical Education and Sport Sciences, Faculty of Humanities and Social Sciences, University of Kurdistan, 66177-15175 Sanandaj, Kurdistan, Iran; saedsaber384@gmail.com; 4 Research Center for High Performance Sport, Campus de los Jerónimos, Catholic University of Murcia, Guadalupe, 30107 Murcia, Spain; lhchung@ucam.edu; 5 Faculty of Sport Sciences, Waseda University, Tokorozawa 359-1192, Japan; katsu.suzu@waseda.jp; 6 Department of Physiology, School of Sport Sciences, University of Extremadura, 10003 Caceres, Spain; mmaynar@unex.es

**Keywords:** antioxidant status, nutrition, performance, physical activity, reactive oxygen species (ROS)

## Abstract

Free radicals are reactive products that have multiple effects on the human body. Endogenous and exogenous antioxidants manage the overproduction of free radicals. However, an imbalance between free radicals and antioxidant factors causes oxidative stress. Exercise and physical activity are factors that increase oxidative stress and disrupts the body’s homeostasis. Intensity and duration of training, training characteristics, and fitness level can have positive or negative effects on oxidative stress. Green tea consumption is recommended for the prevention of a variety of diseases, health maintenance, and weight loss. The effectiveness of green tea is primarily due to the presence of catechins and polyphenols, specifically (–)-epigallocatechin-3-gallate, which has antioxidant and anti-inflammatory properties based on clinical and animal studies. This review investigates the effect of green tea exercise and their interactive effects on free radicals and sports improvement.

## 1. Introduction 

Regular exercise is fundamental for maintaining a healthy lifestyle. Specifically, >150 min of moderate-intensity aerobic activity or >75 min of intense physical activity (PA) per week is recommended for cardiovascular health and optimal function of body organs, while 300 min of moderate-intensity exercise per week is recommended to lower the risk of cancer [1]. These recommendations are based on many systematic reviews and meta-analyses of epidemiological studies [2]. However, exercise has both positive and negative effects on the inflammatory status, oxidative stress, and function of body organs. While moderate-intensity exercise improves immune function as opposed to sedentary behavior, immune system activity can be harmed by prolonged and intense exercise [3,4]. Biomarkers, such as lipid peroxidation, oxidized glutathione, and total radical-trapping antioxidant parameter, have important roles in health and body function as well as during exercise [4]. However, there is limited research regarding the effect of exercise on immune system activity and oxidative stress [5]. 

Exercise is commonly divided into aerobic and resistance training. Aerobic exercise (e.g., running, walking, swimming, and cycling) improves the cardiovascular system and significantly reduces reactive oxygen species (ROS) and various ROS-related reactions. Aerobic exercise increases the presence of antioxidants and improves the expression of antioxidant enzymes, thereby counteracting exercise-induced oxidative stress [6,7]. On the other hand, resistance training provokes an increase in oxidative and inflammatory stress despite promoting neural and structural (i.e., muscle fibers) adaptations. The rest period lowers oxidative stress, where there is reparation of minor injuries induced by resistance training due to increased levels of oxidative and inflammatory stressors. Strenuous physical exercise can stimulate high production of ROS and reactive nitrogen species (RNS) in skeletal muscle. Depending on the exercise level, duration, frequency, sex, age, and fitness, high concentration of reactive species can be harmful to the body if it is not neutralized by the available antioxidant enzymes [8]. Nevertheless, the adaptations that occur from regular and intense exercise reduce overall oxidative stress over time [8].

An imbalance between oxidants and the production of free radicals in the body with the body’s antioxidant status leads to an increase in ROS, which ultimately leads to oxidative stress [9]. Oxidative stress induced by physical activity can reduce the effectiveness of endogenous antioxidants and may lower athletic performance [10]. However, the consumption of rich sources of antioxidants (such as polyphenols and flavonoids) can decrease oxidative stress levels [10]. One source of antioxidants is tea, which is the second most consumed beverage in the world after water [11]. Evidence shows that tea was first used in China around 2737 BC not only as a beverage but also for medicinal purposes [12]. There are three types of tea found in nature: green tea, oolong tea, and black tea. The elements of tea are mainly polyphenols, caffeine, and minerals, along with small amounts of vitamins, amino acids, and carbohydrates. The polyphenol that predominates in tea varies depending on the type of tea. For example, catechin is the major polyphenol in green tea, while tannin is the major polyphenol in black tea [12]. These natural substances have antioxidant properties with very limited side effects. Green tea has been shown to significantly reduce free radicals and oxidative stress [12], which will be discussed in more detail in the following sections. Therefore, green tea consumption together with physical activity can be more effective in eliminating or reducing oxidative stress than drinking green tea or doing exercise alone. Given the wide range of signaling pathways involved with exercise and tea consumption, this overview will only address oxidative stress, the antioxidant effectiveness of green tea and exercise, and their interaction.

## 2. Production of Free Radical and oxidants

ROS are metabolic by-products that include superoxide (−O_2_), hydrogen peroxide (H_2_O_2_), hydroxyl (−OH), and single oxygen (−O_2_) radicals. Protein phosphorylation, activation of multiple transcription factors, apoptosis, immunity, and differentiation require a certain amount of ROS production, which is produced by the cell at low levels. However, overproduction of ROS can damage important structures within the cell, such as proteins, lipids, and nucleic acids [13,14]. The mitochondria are the primary organelles that produce ROS, but endothelial and inflammatory cells can also produce ROS because of cellular respiration under physiological and pathological conditions [13,14]. Although these cells have a special capacity to produce intrinsic ROS [15,16], they cannot reach the same quantity as mitochondrial ROS [15]. ROS production is dependent on enzymatic and nonenzymatic reactions. Enzymatic reactions, such as respiratory chain reactions, are capable of producing ROS [15]. Nonenzymatic reactions involve oxygen reacting with organic matter, thereby yielding free radicals [15]. The production of free radicals can be endogenous or exogenous. The endogenous pathway can result from immune cell activation, inflammation, ischemia, infection, cancer, excessive exercise, mental stress, and aging, while the exogenous pathway can arise from external factors, such as pollution, smoking, drugs, etc.

## 3. Physiological Levels of Reactive Oxygen Species (ROS)

At physiological levels, ROS performs a variety of beneficial body functions. For example, ROS is needed for the synthesis of certain cellular structures and is required by the host defense immune system to counteract external factors. Specifically, phagocytes synthesize and store free radicals to be released when exposed to pathogens. Free radicals play an important regulatory role in cellular signaling pathways and have a function in certain cell types, such as fibroblasts, endothelial cells, vascular smooth muscle cells, cardiac myocytes, and thyroid tissue [17]. In addition, the production of free radicals is physiologically beneficial to human health, such as for patients with granulomatous disease who cannot produce O_2_. At the physiological level, mitochondrial ROS, especially superoxide anion and hydrogen peroxide, play a positive role in responding to factors such as vascular shear stress and reducing vascular resistance to blood flow [18]. Assessing the availability of oxygen is very important for cellular health because it allows cells to initiate adaptive reactions to survive and access oxygen. Schumacker observed that the electron transfers chain acts as an O_2_ deficiency marker by releasing ROS in response to hypoxia, which in turn initiates a signaling mechanism to react appropriately to this process, such as by increasing production and stabilizing HIF-1 [19]. In the physiological state of ROS production, skeletal muscle can also be a target organ for regulating oxidation and oxidative stress. Because muscles need a lot of energy during exercise, this process can increase mitochondrial ROS production. Several pathways can increase ROS production in skeletal muscle, including muscle contraction, insulin, and hypoxia. For example, ROS can be a signal mediator in regulating skeletal muscle glucose uptake during muscle activity [18].

## 4. Overproduction of Free Radicals in the Body: Role of Mitochondria in Oxidative Stress Production and Immune System Response

The overproduction of free radicals and oxidants (i.e., nonphysiological level) causes oxidative stress, which can be responsible for several pathological diseases that affect different tissues and organs and is one of the underlying factors that can be harmful to overall health. This lack of balance between free radical production and antioxidant neutralization can consequently be harmful to body tissues (e.g., cell membranes, lipids, proteins, lipoproteins, and deoxyribonucleic acid structures) and can activate destructive mechanisms [20]. For example, the overproduction of –OH radicals and peroxynitrite leads to lipid peroxidation, which in turn damages the cell membrane. Families of ROS, such as single oxygen, superoxide anion (O_2^−^_), H_2_O_2_, peroxyl radical (i.e., ROO), and –OH radical, are highly reactive. Nitrogen-containing active species, such as nitric oxide (NO) and peroxynitrite anion (ONOO), are also present. The stimulants of active species are radiation, drugs, xenobiotics, and toxins. In addition, exercise can produce free radicals if the intensity, duration, or volume of training is high, which can damage the inner membrane of the mitochondria (where aerobic respiration takes place) and disrupt cell homeostasis [5]. 

Mitochondria are complex organelles with a bilayer membrane, and any dysfunction in this organelle can trigger small oxidative stress signals. As mitochondria primarily function to generate energy and release various ions, they are in turn vulnerable to these charged ions. For example, high production of free radicals during mitochondrial deoxyribonucleic acid (DNA) damage causes an increase in oxidative stress on the mitochondria and can consequently damage it. Recently, thioredoxin 2 (Trx2), a low-redox protein in the mitochondria with two redox regions (C90 and C93), has attracted the attention of researchers [21]. Trx2 is found almost exclusively in tissues with high metabolic levels, such as liver, brain, and heart. Trx2 helps maintain intracellular stability through reversible oxidation of disulfide. It also regulates the function of many apoptosis-related factors, such as apoptosis signal regulator kinase 1 (ASK1) and nuclear factor kappa B (NF-κB) [22]. Low levels of Trx2 expression causes the release of cytochrome c from the mitochondria, followed by activation of caspase-3 and -9. Furthermore, Trx2 reduces mitochondrial reactive oxygen species (mctROS) and tumor necrosis factor (TNF)-dependent apoptosis. Thus, low-redox mitochondrial protein Trx2 is a regulator of oxidative stress.

Research has shown that mitochondria can regulate the response of immune cells via oxidative stress reactions [23]. For example, the mitochondrial process regulates the function of memory T cells [24,25]. ROS are an important factor in stimulating the immune system [8]. When responding to oxidative stress, the immune system produces factors that consequently cause oxidative stress. This means active species do not recognize their own factors, so the immune-response-induced oxidative stress may also attack their own factors, creating a vicious cycle. However, the immune system also fights against the oxidative stress agent by activating its complex antioxidant system (which include enzymes, minerals, and vitamins) to neutralize the reactive species [26]. Reactive active species release proinflammatory cytokines, TNF-alpha (TNF-α) and interleukins, which eventually initiate an inflammatory response. Accumulation of these inflammatory factors releases the body’s defense factors, such as interferon-gamma (IFN-γ), cluster of differentiation 14 (CD14), or TNF-α. In addition, the immune response produces an increase in energy and physiological costs, an example of the latter being a fever, which is produced by cytokine activity. A fever of above two degrees Fahrenheit has a metabolic cost of 175 KJ in a person. Thus, this can delay recovery after exercise [27,28]. Regardless, the endogenous antioxidant defense system eliminates these active species. Furthermore, consuming exogenous antioxidant supplements can help maintain proper immune system function [29].

## 5. Neutralizing Free Radicals: Green Tea

Natural interventions to reduce the negative effects of exercise and improve body function during exercise has found its place in the athletes’ training program. The use of supplements is often promoted to athletes with claims of improving performance, but it may not clearly specify their ergogenic effects. Thus, extensive research is being done on the role of their effectiveness on sports performance [30]. 

Among dietary and natural supplements, tea is popular and widely used due to its antioxidant, anti-inflammatory, and anticarcinogenic properties [29]. Among them, black tea is highly consumed (78% popularity), followed by green tea (20% popularity) [31]. Green tea is a very potent beverage because of its antioxidant, anti-inflammatory, anticarcinogenic, and antiallergic properties [32]. Additionally, green tea decreases the symptoms of metabolic syndrome and diabetes by controlling blood glucose, lowering cholesterol, etc. [31].

### 5.1. Green Tea Chemical Composition and Its Biological Characteristics

The chemical formula of green tea consists of proteins and amino acids (20–25% raw material), such as glutamic acid, tryptophan, glycine, serine, aspartic acid, tyrosine, valine, leucine, threonine, arginine, sinus, and carbohydrates (5–7% raw material; cellulose, glucose, fructose, and sucrose). It also contains lipids (nickel linoleic acid and linoleic acid), vitamins (B, C, and E), caffeine, chlorophylls, and carotenoids. Unstable compounds (aldehydes, alcohols, esters, lactones, and hydrocarbons), minerals, and essential elements (5% dry weight; Ca, Mg, Cr, Mn, Fe, Cu, Zn, Mo, Se, Na, P, Co, Sr, Ni, K, F, and Al) are also included. Green tea has a rich source of polyphenols, such as flavonoids. Flavonoids are phenolic derivatives that differ in their concentration in green tea [33]. Catechins are the most important flavonoids in green tea. 

The components in green tea have medicinal properties because of the presence of polyphenols, specifically flavonoids. These flavonoids contain a high proportion of catechins (80–90%) compared to other teas. Green tea has four main catechins: epigallocatechin gallate (EGCG; 60%), epigallocatechin (EGC; 20%), epicatechin-3-gallate (ECG; 14%) and epicatechin (EC; 6%). Among them, EGCG has the most health benefits, as it is effective in maintaining cardiovascular health and metabolism. In green tea, the amount of catechins varies, although a standard extract has been acquired for its use in supplementation [31]. Catechins cannot be completely extracted from fresh green tea leaves; therefore, there are large differences in the extract concentrations obtained. In addition, catechins are relatively unstable and can vary in amount under different conditions. Therefore, it is not possible to estimate the doses of catechins used in animal studies due to their lack of quantification [10,31,34].

The metabolic reactions for all catechins follow the same pathways of phase II detoxification reactions. Animal studies show that catechin uptake occurs in the intestine and liver [35]. Catechins are partially bound to intestinal mucosa, liver, and kidneys, and about 5% of catechins are transported freely (i.e., unbound) through the bloodstream. For optimal biochemical functioning, catechins must attach to certain biochemical components (e.g., glutathione via liver enzymes) to improve their water solubility and facilitate their excretion in bile and urine. Thus, large amounts of catechins (including bile-attached catechins) are not absorbed in the small intestine and are transported to the large intestine, where they are broken down to smaller metabolites by resident microbes and reabsorbed for excretion via the urine as valerolactones [36]. Catechins were initially thought to have low viability of about 8–9% and very high detoxification [37]. However, studies have observed that the bioavailability of catechins increases by about 40% due to antimicrobial activity [38,39], where catechins were present up to 48 h after ingestion in human urinary excretion. This increase in bioavailability of catechins is explained by the conversion of larger compounds (ECGC and ECG) to smaller compounds (such as UDP-glucuronosltransferases and sulphotransferases) via the phase II detoxification reaction.

### 5.2. Effectiveness and Life Span of Green Tea Catechins

Pharmacological research demonstrates that green tea catechins have an effectiveness in the range of 2–13% in mice [40]. Some properties that can contribute to the oral bioavailability of green tea include its low solubility in gastrointestinal fluid, low permeability of the membrane, its degradation and metabolism in the gastrointestinal tract, and its transmission through intestinal epithelial membrane. Green tea catechins are stable at pH <6.5, but EGC and EGCG are rapidly degraded at pH >7.4. The concentration of polyphenols is higher in the fasted stated than at postprandial [40]. Isbrucker et al. found that 20 mg·kg^−1^ body mass per day of EGCG had no side effects in animals [41]. Most animal studies have used 0.1% green tea extract, equivalent to 10 mg per 100 mL of dissolved water, because it showed effectiveness with the lower amount [41].

Gallate-type catechins (e.g., EGCG and ECG) have a longer life span than nongalactic catechins (EGC and EC) because they are bound to proteins, which prevent premature excretion. The short half-life of nongalactic catechins occurs when it crosses the intestinal wall. The lasting effect of the maximum serum level of green tea catechins is about one hour in the fasted state and about two hours in the satiated state [42]. Green tea catechins are shown to be more stable at low pH and in the fasted state [43]. Alimentation raises the pH of the gut, a condition that destroys catechins. 

### 5.3. Antioxidant Function Green Tea 

**Figure 1:**
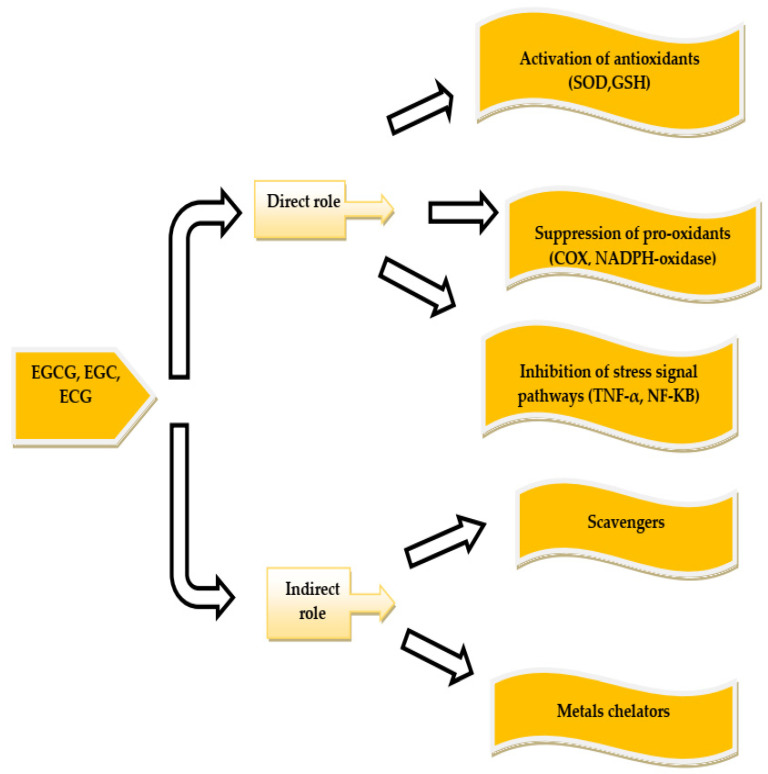
Mechanism regarding the effect of green tea catechins on oxidative stress.

Green tea is a rich source of antioxidants. Structural properties of green tea catechins play a role in their antioxidant function. The presence or absence of the galvanic portion and the number and position of the –OH groups on the ring define their structural property. The presence of the carboxyl group enables catechins to interact with the biological substance and gives its antioxidant function by binding to hydrogen or transferring electrons and hydrogen, or both [44]. The antioxidant activity of tea appears to inhibit lipid-inhibiting alkoxyl and peroxyl radicals [45]. For example, the antioxidant activity of green tea extract is due to the increase in superoxide dismutase activity and catalase expression. These enzymes protect cells against oxidative stress [46]. The proposed mechanism for this process is the reduction of nitric oxide concentration in the plasma, which directly lowers the activity of oxygen species [13,47]. Malondialdehyde, a marker of oxidative stress, also decreases after consuming green tea [13,47]. Elevated levels of Phase II antioxidant enzymes have been observed after drinking green tea by increasing levels of polyphenols in the small intestine, lungs, and skin of mice [48] and rat prostate [49] and the oral cavity of hamsters [50]. The underlying mechanism of this process can be through the activation of MAPKs by green tea polyphenols [51]. Activation of the Nrf2 signaling pathway is also involved in this process [52]. Stimulation of phase II enzymes not only neutralizes free radicals and oxidative stress but can also have a detoxifying effect on carcinogens, such as aflatoxin B1 [52]. Studies have shown that catechins have a direct (antioxidant) or indirect (increased activity or expression) effect on oxidative stress. The following Figure 1 shows the effect of green tea catechins on oxidative stress [45].

### 5.4. Green Tea Used as an Ergogenic Aid 

Green tea is an ergogenic supplement that optimizes mitochondrial function and increases lipolysis and fatty acid metabolism, thereby reducing fatigue and improving endurance performance. After a Japanese study examined positive effects of catechins in rats [45], there was an increase in investigations regarding the effects of green tea catechins in animal models [45,46]. One study observed that mice who exercised with consumption of green tea extract (GTE; 0.2% and 0.5% supplementation groups) improved exercise performance compared to the exercise only and control groups, suggesting that the presence of catechin EGCG stimulated lipid metabolism [46]. 

## 6. Sport Performance and Drinking Green Tea

In recent years, many studies have studied the effect of polyphenols, especially catechins, on athletic performance. Most of these studies have examined the effect of catechins on exercise-induced muscle damage and their biological and physiological role in improving physical function. Until a few years ago, as catechins are antioxidants, their role in preventing muscle damage was studied [47]. However, in recent years, more studies have significantly examined the effect of polyphenols on athletic performance. Studies on polyphenols and exercise include antioxidant supplements, such as green tea extract (GTE). Although the majority of studies on green tea have been done on animals, lately, a large number of studies have been done on human athletic performance. Green tea extract contains a large amount of catechins that increase daily energy consumption in humans. For example, short-term consumption of green tea extract on healthy untrained men increased the amount of energy available during 30 min of cycling at 60% of the maximum oxygen consumption. However, the chemical process of green tea and green tea extract in the face of oxidative stress remains unknown. It has been reported that the inhibitory capacity of catechins is due to the presence of a hydroxyl group at the 5 prim position, which increases their ability to inhibit free radicals [47].

## 7. Production ROS during Exercise Training and Its Consequences

**Figure 2:**
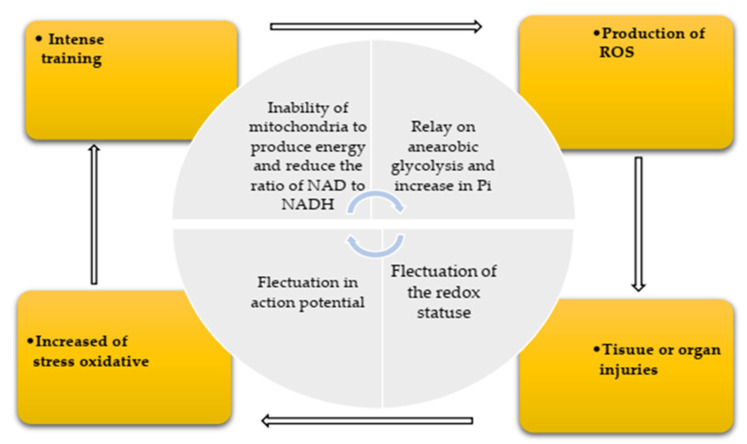
Production of reactive oxygen species (ROS) during exercise training.

There are physiological factors that cause oxidative stress, but the effect of exercise on oxidative balance is difficult because it depends on many factors such as sex, age, and exercise status as well as intensity, duration, and frequency of exercise. Aging and exercise are commonly known as oxidative stressors [48,49]. Research has shown that metabolism, oxygen consumption, and production of active species due to intense exercise are 20 times higher than at rest [50]. Muscle contractions promotes the production of oxidants, which has importance in physiological function of the body. However, prolonged or intense exercise can lead to oxidative damage to cell compounds, and exercise-induced oxidative stress has been shown to affect muscle function [50]. Figure 2 illustrates the effect of exercise on the production of reactive active species.

Numerous studies have shown an increase in oxidative stress factors after chronic aerobic and strenuous exercise [50,51]. Exhausting strenuous exercise provides a good model for assessing the effects of oxidative stress on the body [50]. It is well known that intense and prolonged exercise elicits both lipid peroxidation and muscle damage rather than increasing the capacity of antioxidants [50]. However, some studies have observed that endurance training has no significant effect on inflammatory markers [52]. Nevertheless, research studies have shown that intense, moderate, and long-term aerobic exercise produce free radicals and oxidative stress [52]. Free radicals cause damage to lipids, proteins, carbohydrates, and DNA, among others [53]. Under normal conditions, about 2–5% of oxygen is converted to free radicals by organs.

## 8. Green Tea and Prevention of Oxidative Stress Production during Exercise

In general, the body can neutralize exercise-induced oxidative stress through antioxidant defense [20], but this defense can become overwhelmed by exercise-induced ROS production. The oxidative stress produced in the body is neutralized through internal and external antioxidant systems. Because intense, irregular, and prolonged PA increases energy requirements in body tissues, especially in active muscles, oxygen consumption is greater and enzymatic antioxidant defense systems cannot cope with the higher production of free radicals alone [54]. Thus, nonenzymatic antioxidant system, in the form of natural and oral supplements, can help combat oxidative stress [54]. Consumption of antioxidants, such as green tea polyphenols and catechins, improves the body’s antioxidant status and reduces the damaging effects of radicals during exercise, although these supplements do not appear to have a direct beneficial effect on performance [55,56].

Alessio was the first to examine the effects of green tea catechins on exercise-induced oxidative stress [57]. Five to six weeks of green tea consumption increased the athlete’s antioxidant capacity, which in turn increased the level of total antioxidants in plasma during and after an intense running exercise session as well as prevented or inhibited excessive lipid peroxidation caused by the exercise. Several studies have shown that green tea has a protective effect (i.e., increased plasma antioxidant and decreased plasma lipid hydroperoxide levels) against oxidative stress produced during and after exercise [58,59]. In addition, green tea consumption inhibits or reduces plasma creatine kinase and xanthine oxidase activities after exercise. Numerous studies have shown that long-term use of antioxidant supplements lowers the production of free radicals and can diminish or even inhibit signaling from reactive species that are harmful to the body [60].

On the other hand, research has shown that exercise activates the redox-sensitive transcription factor NF-κB, which retranscribes the expression of antioxidant factor genes [50]. NF-κB is the main stimulus in inflammation and the main element of the innate immune response [45]. In addition, it plays an essential role in adaptive immune responses and regulates embryonic development, lymphopoiesis, and osteogenesis [61]. NF-κB is a redox-sensitive transcription factor that is overproduced and proposed to be involved in the regulation of cellular activity, including inflammation, immune response, growth, and cell death [61]. NF-κB remains in the cytoplasm as long as the kappa B inhibitor (IKB) 2 is inactive, but it is transported to the nucleus when IKB is activated [61]. EGCG reduces NF-κB binding to the nucleus and reduces the expression of transcription factor P65, the NF-κB subunit, by TNF-α [31]. Exercise and muscle contractions stimulate the sarcoplasmic release of calcium, increase ROS, and activate numerous signaling cascades, such as mitogen-activated protein kinase (MAPK) [31]. Various cell samples have been shown to increase intracellular calcium, ROS accumulation, and MAPK activation to enable NF-κB, suggesting that exercise can also activate NF-κB [52]. Acute treadmill exercise increases IκB kinase α and β (IKKα/β) phosphorylation, nuclear factor of kappa light polypeptide gene enhancer in B-cells inhibitor, alpha (IκBα) phosphorylation, and NF-κB activity in rat skeletal muscle [39]. While IκBα phosphorylation level increases during exercise, nuclear-bound NF-κB activity peaks at postexercise intervals [7]. NF-κB activation is a local event in contracting muscle as this process can occur in the absence of exercise-induced systemic factors [60]. Drug inhibitors p38 and extracellular signal-regulated kinase 1/2 (ERK1/2) are able to slow IKKα/β-regulated muscle contraction by 39% and 35%, respectively and by 76% in combination [62]. Alieso et al. showed that green tea EGCG protects the kidneys against lipid peroxidation [57]. However, Pingitore et al. demonstrated that the use of antioxidant supplements can have negative effects on the normal function of athletes, where low levels of oxidative stress reduce the positive reactions associated with hormones because some free radicals play an important role in signaling processes related to cell function [20].

**Figure 3:**
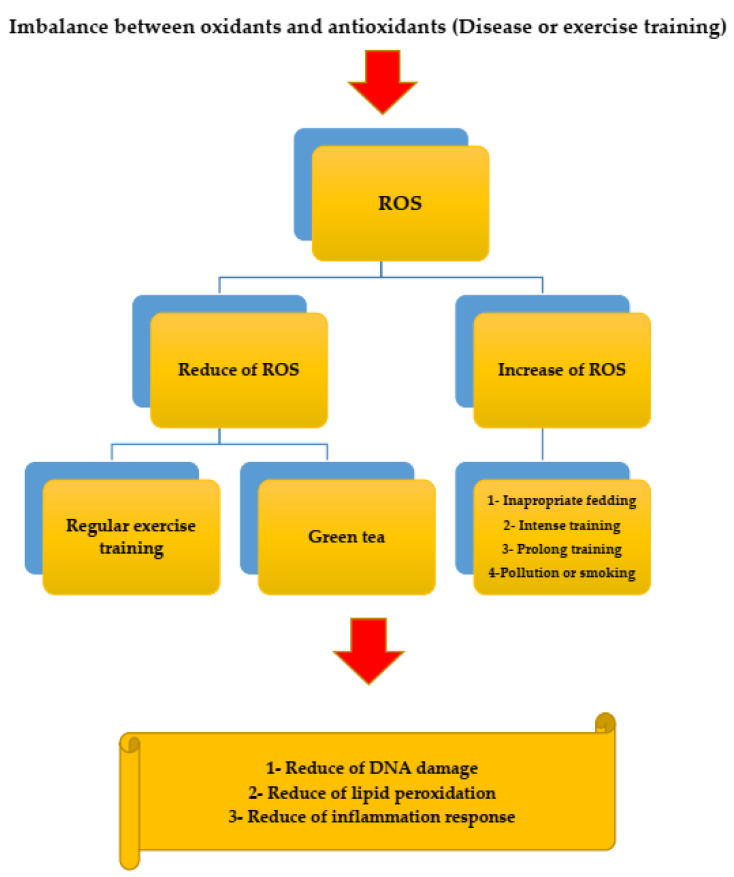
The relationship between green tea and reactive oxygen species (ROS).

Tea polyphenols act as antioxidants by reducing or inhibiting ROS and nitrogen through accumulating active metal ions, and they may indirectly act as antioxidants through other mechanisms that inhibit enzymes. “Pro-oxidant” and induced antioxidant enzymes protect tissues, cells, and plasma compounds against oxidative damage (see Figure 3).

## 9. How Can Green Tea Improve Sports Performance?

The use of natural strategies and interventions to improve athletic performance, in addition to overall health, has attracted the attention of athletes at all levels of sport, and green tea consumption has particularly been demonstrated to be effective [9,36]. Meta-analysis studies have shown that green tea consumption improves performance without any side effects [25,34,36]. Further meta-analysis investigations on antioxidant effects and the presence of polyphenols in green tea have shown its beneficial effects on exercise performance [36,42]. There are many mechanisms for the effectiveness of polyphenols. Although it is not possible to address them all here, we will mention some of them [63]. Natural interventions, such as plant extracts and phytochemicals, enhance physical function, improve recovery after exercise, maintain overall health [35], and have minimal side effects [64,65]. In addition, regular green tea consumption (434 mL·day^−1^) was found to reduce body fat and decrease waist-to-pelvis ratio compared to the control group (i.e., no green tea ingestion). Similar to these findings, rodent models with high fat diet and body fat mass lost weight with green tea catechin consumption [66,67]. 

**Figure 4:**
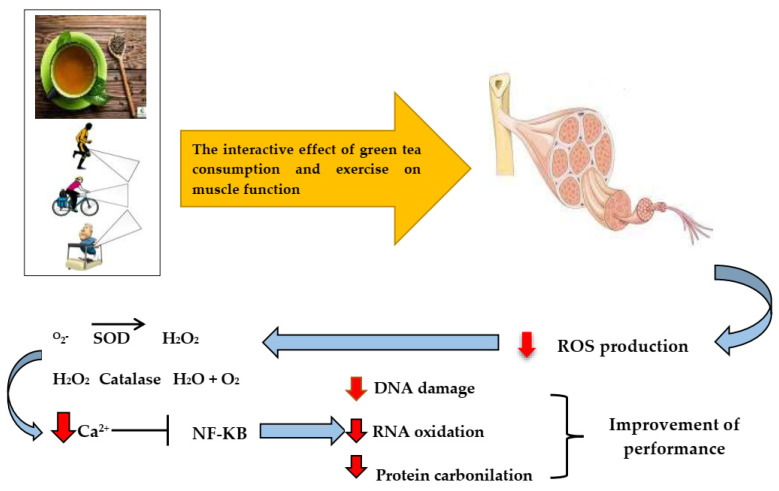
Possible mechanisms related to the effects of green tea catechins and exercise training on performance. Exercise and concomitant consumption of green tea reduce oxidative factors and suppress the activation of inflammatory factors by regulating calcium release, reduce DNA and RNA damage, release caspases due to TRX activation, and ultimately lead to improved performance.

Green tea polyphenols and catechins also affect SIRT1, which has a role in increasing the activity of PGC-1a after deacetylation, consequently improving mitochondrial function [68,69]. Another action of green tea polyphenols and catechins is increased endothelial NO synthesis and vasodilation [70,71]. The production of NO improves the perfusion of oxygen to the active muscles and improves athletic performance [72,73]. Thus, GTE supplementation can reduce oxidative stress through its polyphenols [74] and produce NO to improve maximum oxygen uptake, thus delaying fatigue [74]. Additionally, green tea consumption reduces muscle pain caused by improper exercise, bruising, and subsequent injuries. The reduction of exercise-induced fatigue because of GTE supplementation has great practical applications in sports performance in both amateur [75] and professional [52] athletes because it may be the limiting factor in achieving a personal record or successful performance. Oxidative stress not only accumulates during exercise [75] but also does so after exercise; thus, the consumption of green tea during and following exercise can lower oxidative stress (i.e., lipid peroxidation) [76]. Plasma triglyceride levels can indicate the effectiveness of catechins, which has been shown to be reduced with exercise in normal mice [77]. In addition, the consumption of green tea extract lowered triglyceride levels in Zucker mice and mice fed with a sucrose-rich diet. Studies have demonstrated that green tea flavonoids have an insulin-like activity and increase insulin activity [77]. The Figure 4 shows the effect of exercise combined with green tea consumption on exercise performance.

Ichinose et al. examined the effect of drinking green tea with moderate-intensity exercise and found that concomitant use of the two interventions increased metabolism of fat, which was the predominant fuel during exercise [78]. The predominance of the fat energy source reduced VO_2_ during exercise. This process resulted in a 10% reduction in post-workout oxygen consumption in both groups, which was consistent with other studies [79,80]. They found that exercise combined with drinking green tea resulted in metabolic adaptation and reduced energy expenditure at the same intensity of exercise. This reduction in energy leads to a decrease in the amount of basal metabolism. They observed this mechanism by measuring the concentration of free fatty acids after exercise and reported that the level of free fatty acids was significantly higher immediately after exercise in trained mice. Beta-oxidation cycle enzymes are important during exercise and fatty acid oxidation [81]. Murase et al. [82] found that drinking tea with exercise improved the activity of beta-oxidation enzymes and improved mitochondrial enzymes. They also found that drinking tea with exercise delayed the onset of fatigue [82]. According to studies and observation of the interaction between exercise and nutritional interventions, new insights can be provided for the use of natural interventions during physical activity to improve athletic performance or maintain health.

One of the limitations of our review is that there is a wide range of oxidative stress factors that could not be addressed. Additionally, this review only mentioned few of the many effects of green tea on performance and health. The amount of green tea required to obtain the maximum benefits is also unclear. Moreover, it is not clear how much physiological oxidative stress production is beneficial and how much harms the body. Most of the research has been done on healthy and young people, and there have been few investigations examining oxidative stress factors associated with metabolic syndrome and other diseases. Therefore, more studies are needed to answer these questions.

## 10. Conclusions

Due to its many properties, green tea improves physical and physiological function of the body during exercise by diminishing oxidative stress; however, more research is still needed. Assuming that green tea improves antioxidant function in active muscles while performing strength and endurance training, further studies should examine different training models to better understand its benefits on athletic performance. Due to the fat burning, weight loss, and mitochondrial function of green tea, most studies of exercise protocols associated with green tea have used aerobic exercise protocols. Due to the properties of green tea in changing body composition, weight loss, maintaining lean body mass, reducing body fat, reducing waist circumference, and reducing body fat percentage, green tea consumption combined with resistance training exercises causes more changes in anthropometric characteristics compared to strength training alone. Therefore, much research is needed on the effectiveness of different exercise models with green tea supplementation in physiological contexts and signaling and molecular pathways.

## Data Availability

Not applicable.

## References

[1] Ruegsegger G.N., Booth F.W. (2018). Health benefits of exercise. Cold Spring Harb. Perspect. Med..

[2] Füzéki E., Banzer W. (2018). Physical activity recommendations for health and beyond in currently inactive populations. Int. J. Environ. Res. Public Health.

[3] Abd El-Kader S.M., Al-Shreef F.M. (2018). Inflammatory cytokines and immune system modulation by aerobic versus resisted exercise training for elderly. Afr. Health Sci..

[4] Jones A.W., Davison G. (2019). Exercise, immunity, and illness. Muscle and Exercise Physiology.

[5] Nobari H., Nejad H.A., Kargarfard M., Mohseni S., Suzuki K., Carmelo Adsuar J., Pérez-Gómez J. (2021). The Effect of Acute Intense Exercise on Activity of Antioxidant Enzymes in Smokers and Non-smokers. Biomolecules.

[6] Radak Z., Ishihara K., Tekus E., Varga C., Posa A., Balogh L., Boldogh I., Koltai E. (2017). Exercise, oxidants, and antioxidants change the shape of the bell-shaped hormesis curve. Redox Biol..

[7] Thirupathi A., Wang M., Lin J.K., Fekete G., István B., Baker J.S., Gu Y. (2021). Effect of Different Exercise Modalities on Oxidative Stress: A Systematic Review. BioMed Res. Int..

[8] Orlando P., Silvestri S., Galeazzi R., Antonicelli R., Marcheggiani F., Cirilli I., Bacchetti T., Tiano L. (2018). Effect of ubiquinol supplementation on biochemical and oxidative stress indexes after intense exercise in young athletes. Redox Rep..

[9] Cabrera C., Artacho R., Giménez R. (2006). Beneficial effects of green tea—A review. J. Am. Coll. Nutr..

[10] Vahabzadeh Z., Molodi M., Nikkho B., Saghebjoo M., Saedmocheshi S., Zamani F., Roshani Y., Babanzadeh S. (2020). Aerobic training and hydroalcoholic extracts of green tea improve pro-oxidant-antioxidant balance and histopathological score in the N-methyl-N-nitrosourea-induced prostate cancer model of rat. EXCLI J..

[11] Vayalil P.K., Mittal A., Hara Y., Elmets C.A., Katiyar S.K. (2004). Green tea polyphenols prevent ultraviolet light-induced oxidative damage and matrix metalloproteinases expression in mouse skin. J. Investig. Dermatol..

[12] Prasanth M.I., Sivamaruthi B.S., Chaiyasut C., Tencomnao T. (2019). A review of the role of green tea (Camellia sinensis) in antiphotoaging, stress resistance, neuroprotection, and autophagy. Nutrients.

[13] Centner C., Zdzieblik D., Dressler P., Fink B., Gollhofer A., Koenig D. (2018). Acute effects of blood flow restriction on exercise-induced free radical production in young and healthy subjects. Free Radic. Res..

[14] Bouzid M.A., Filaire E., McCall A., Fabre C. (2015). Radical oxygen species, exercise and aging: An update. Sports Med..

[15] Hart C.R., Layec G., Trinity J.D., Kwon O.S., Zhao J., Reese V.R., Gifford J.R., Richardson R.S. (2018). Increased skeletal muscle mitochondrial free radical production in peripheral arterial disease despite preserved mitochondrial respiratory capacity. Exp. Physiol..

[16] Lushchak V.I. (2014). Free radicals, reactive oxygen species, oxidative stress and its classification. Chem.-Biol. Interact..

[17] Ranneh Y., Ali F., Akim A.M., Hamid H.A., Khazaai H., Fadel A. (2017). Crosstalk between reactive oxygen species and pro-inflammatory markers in developing various chronic diseases: A review. Appl. Biol. Chem..

[18] Halliwell B., Gutteridge J.C., Cross C.E. (1992). Free radicals, antioxidants, and human disease: Where are we now?. J. Lab. Clin. Med..

[19] Schumacker P.T. (2003). Current paradigms in cellular oxygen sensing. Hypoxia.

[20] Pingitore A., Lima G.P.P., Mastorci F., Quinones A., Iervasi G., Vassalle C. (2015). Exercise and oxidative stress: Potential effects of antioxidant dietary strategies in sports. Nutrition.

[21] Patenaude A., Murthy M.V., Mirault M.-E. (2004). Mitochondrial thioredoxin system: Effects of TrxR2 overexpression on redox balance, cell growth, and apoptosis. J. Biol. Chem..

[22] Hansen J.M., Go Y.-M., Jones D.P. (2006). Nuclear and mitochondrial compartmentation of oxidative stress and redox signaling. Annu. Rev. Pharmacol. Toxicol..

[23] Sastre J., Pallardó F.V., García de la Asunción J., Viña J. (2000). Mitochondria, oxidative stress and aging. Free Radic. Res..

[24] Chen Y., Zhou Z., Min W. (2018). Mitochondria, oxidative stress and innate immunity. Front. Physiol..

[25] Zhou Y., Huang L., Zheng W., An J., Zhan Z., Wang L., Chen Z., Liu L. (2018). Recurrent nonsevere hypoglycemia exacerbates imbalance of mitochondrial homeostasis leading to synapse injury and cognitive deficit in diabetes. Am. J. Physiol.-Endocrinol. Metab..

[26] Somani S.M., Husain K., Schlorff E.C. (2017). Response of antioxidant system to physical and chemical stress. Oxidants, Antioxidants, and Free Radicals.

[27] Jacot A., Scheuber H., Brinkhof M.W. (2004). Costs of an induced immune response on sexual display and longevity in field crickets. Evolution.

[28] Costantini D., Møller A.P. (2009). Does immune response cause oxidative stress in birds? A meta-analysis. Comp. Biochem. Physiol. Part A Mol. Integr. Physiol..

[29] Gleeson M., Nieman D.C., Pedersen B.K. (2004). Exercise, nutrition and immune function. J. Sports Sci..

[30] Medicine A.C.o.S., Association A.D. (2000). Joint Position Statement: Nutrition and athletic performance. American College of Sports Medicine, American Dietetic Association, and Dietitians of Canada. Med. Sci. Sports Exerc..

[31] Saedmocheshi S., Saghebjoo M., Vahabzadeh Z., Sheikholeslami-Vatani D. (2019). Aerobic Training and Green Tea Extract Protect Against N-methyl-N-nitrosourea-induced Prostate Cancer. Med. Sci. Sports Exerc..

[32] Takahashi M., Miyashita M., Suzuki K., Bae S.-r., Kim H.-K., Wakisaka T., Matsui Y., Takeshita M., Yasunaga K. (2014). Acute ingestion of catechin-rich green tea improves postprandial glucose status and increases serum thioredoxin concentrations in postmenopausal women. Br. J. Nutr..

[33] Musial C., Kuban-Jankowska A., Gorska-Ponikowska M. (2020). Beneficial properties of green tea catechins. Int. J. Mol. Sci..

[34] Khan N., Mukhtar H. (2013). Tea and health: Studies in humans. Curr. Pharm. Des..

[35] Kalogeropoulos N., Chiou A., Ioannou M.S., Karathanos V.T. (2013). Nutritional evaluation and health promoting activities of nuts and seeds cultivated in Greece. Int. J. Food Sci. Nutr..

[36] Higdon J.V., Frei B. (2003). Tea catechins and polyphenols: Health effects, metabolism, and antioxidant functions. Crit. Rev. Food Sci. Nutr..

[37] Kanwar J., Taskeen M., Mohammad I., Huo C., Chan T.H., Dou Q.P. (2012). Recent advances on tea polyphenols. Front. Biosci. (Elite Ed.).

[38] Del Rio D., Calani L., Cordero C., Salvatore S., Pellegrini N., Brighenti F. (2010). Bioavailability and catabolism of green tea flavan-3-ols in humans. Nutrition.

[39] Calani L., Del Rio D., Luisa Callegari M., Morelli L., Brighenti F. (2012). Updated bioavailability and 48 h excretion profile of flavan-3-ols from green tea in humans. Int. J. Food Sci. Nutr..

[40] Peluso I., Serafini M. (2017). Antioxidants from black and green tea: From dietary modulation of oxidative stress to pharmacological mechanisms. Br. J. Pharmacol..

[41] Isbrucker R., Edwards J., Wolz E., Davidovich A., Bausch J. (2006). Safety studies on epigallocatechin gallate (EGCG) preparations. Part 2: Dermal, acute and short-term toxicity studies. Food Chem. Toxicol..

[42] Yang C.S., Wang X. (2010). Green tea and cancer prevention. Nutr. Cancer.

[43] Ananingsih V.K., Sharma A., Zhou W. (2013). Green tea catechins during food processing and storage: A review on stability and detection. Food Res. Int..

[44] Lambert J.D., Elias R.J. (2010). The antioxidant and pro-oxidant activities of green tea polyphenols: A role in cancer prevention. Arch. Biochem. Biophys..

[45] Ikeda I., Kobayashi M., Hamada T., Tsuda K., Goto H., Imaizumi K., Nozawa A., Sugimoto A., Kakuda T. (2003). Heat-epimerized tea catechins rich in gallocatechin gallate and catechin gallate are more effective to inhibit cholesterol absorption than tea catechins rich in epigallocatechin gallate and epicatechin gallate. J. Agric. Food Chem..

[46] Murase T., Haramizu S., Shimotoyodome A., Nagasawa A., Tokimitsu I. (2005). Green tea extract improves endurance capacity and increases muscle lipid oxidation in mice. Am. J. Physiol.-Regul. Integr. Comp. Physiol..

[47] Özyurt H., Luna C., Estévez M. (2016). Redox chemistry of the molecular interactions between tea catechins and human serum proteins under simulated hyperglycemic conditions. Food Funct..

[48] Radak Z., Chung H.Y., Koltai E., Taylor A.W., Goto S. (2008). Exercise, oxidative stress and hormesis. Ageing Res. Rev..

[49] McARDLE A., Jackson M.J. (2000). Exercise, oxidative stress and ageing. J. Anat..

[50] Powers S.K., Ji L.L., Leeuwenburgh C. (1999). Exercise training-induced alterations in skeletal muscle antioxidant capacity: A brief review. Med. Sci. Sports Exerc..

[51] Koubaa A., Triki M., Trabelsi H., Baati H., Sahnoun Z., Hakim A. (2015). The effect of a 12-week moderate intensity interval training program on the antioxidant defense capability and lipid profile in men smoking cigarettes or hookah: A cohort study. Sci. World J..

[52] Mikkelsen U., Couppé C., Karlsen A., Grosset J., Schjerling P., Mackey A., Klausen H., Magnusson S., Kjær M. (2013). Life-long endurance exercise in humans: Circulating levels of inflammatory markers and leg muscle size. Mech. Ageing Dev..

[53] Farhadi H., Siakuhian M., Dolatkhah H., Rahimifardin S., Salemi S.N.P. (2013). Effect of short-term garlic supplementation on DNA damage after exhaustive exercise in non-athlete men. Eur. J. Exp. Biol..

[54] Magherini F., Fiaschi T., Marzocchini R., Mannelli M., Gamberi T., Modesti P.A., Modesti A. (2019). Oxidative stress in exercise training: The involvement of inflammation and peripheral signals. Free Radic. Res..

[55] Hadi A., Pourmasoumi M., Kafeshani M., Karimian J., Maracy M.R., Entezari M.H. (2017). The effect of green tea and sour tea (Hibiscus sabdariffa L.) supplementation on oxidative stress and muscle damage in athletes. J. Diet. Suppl..

[56] Rahimi R., Falahi Z. (2017). Effect of green tea extract on exercise-induced oxidative stress in obese men: A randomized, double-blind, placebo-controlled, crossover study. Asian J. Sports Med..

[57] Alessio H.M., Hagerman A.E., Romanello M., Carando S., Threlkeld M.S., Rogers J., Dimitrova Y., Muhammed S., Wiley R.L. (2002). Consumption of green tea protects rats from exercise-induced oxidative stress in kidney and liver. Nutr. Res..

[58] Jówko E., Płaszewski M., Cieśliński M., Sacewicz T., Cieśliński I., Jarocka M. (2019). The effect of low level laser irradiation on oxidative stress, muscle damage and function following neuromuscular electrical stimulation. A double blind, randomised, crossover trial. BMC Sports Sci. Med. Rehabil..

[59] Panza V.S.P., Wazlawik E., Schütz G.R., Comin L., Hecht K.C., da Silva E.L. (2008). Consumption of green tea favorably affects oxidative stress markers in weight-trained men. Nutrition.

[60] Gomez-Cabrera M.C., Borrás C., Pallardó F.V., Sastre J., Ji L.L., Viña J. (2005). Decreasing xanthine oxidase-mediated oxidative stress prevents useful cellular adaptations to exercise in rats. J. Physiol..

[61] Hellweg C.E. (2015). The nuclear factor κB pathway: A link to the immune system in the radiation response. Cancer Lett..

[62] Vider J., Lehtmaa J., Kullisaar T., Vihalemm T., Zilmer K., Kairane Č., Landor A., Karu T., Zilmer M. (2001). Acute immune response in respect to exercise-induced oxidative stress. Pathophysiology.

[63] Shixian Q., VanCrey B., Shi J., Kakuda Y., Jiang Y. (2006). Green tea extract thermogenesis-induced weight loss by epigallocatechin gallate inhibition of catechol-O-methyltransferase. J. Med. Food.

[64] Kennedy D.O., Wightman E.L. (2011). Herbal extracts and phytochemicals: Plant secondary metabolites and the enhancement of human brain function. Adv. Nutr..

[65] West B.J., Deng S., Isami F., Uwaya A., Jensen C.J. (2018). The potential health benefits of noni juice: A review of human intervention studies. Foods.

[66] Zhang Y., Yu Y., Li X., Meguro S., Hayashi S., Katashima M., Yasumasu T., Wang J., Li K. (2012). Effects of catechin-enriched green tea beverage on visceral fat loss in adults with a high proportion of visceral fat: A double-blind, placebo-controlled, randomized trial. J. Funct. Foods.

[67] Rains T.M., Agarwal S., Maki K.C. (2011). Antiobesity effects of green tea catechins: A mechanistic review. J. Nutr. Biochem..

[68] Gurd B.J. (2011). Deacetylation of PGC-1α by SIRT1: Importance for skeletal muscle function and exercise-induced mitochondrial biogenesis. Appl. Physiol. Nutr. Metab..

[69] Afzalpour M., Ghasemi E., Zarban A. (2017). Effects of 10 weeks of high intensity interval training and green tea supplementation on serum levels of Sirtuin-1 and peroxisome proliferator-activated receptor gamma co-activator 1-alpha in overweight women. Sci. Sports.

[70] Kim J.-a., Formoso G., Li Y., Potenza M.A., Marasciulo F.L., Montagnani M., Quon M.J. (2007). Epigallocatechin gallate, a green tea polyphenol, mediates NO-dependent vasodilation using signaling pathways in vascular endothelium requiring reactive oxygen species and Fyn. J. Biol. Chem..

[71] Ras R.T., Zock P.L., Draijer R. (2011). Tea consumption enhances endothelial-dependent vasodilation; a meta-analysis. PLoS ONE.

[72] Kingwell B.A. (2000). Nitric oxide-mediated metabolic regulation during exercise: Effects of training in health and cardiovascular disease. FASEB J..

[73] Potenza M.A., Marasciulo F.L., Tarquinio M., Tiravanti E., Colantuono G., Federici A., Kim J.-a., Quon M.J., Montagnani M. (2007). EGCG, a green tea polyphenol, improves endothelial function and insulin sensitivity, reduces blood pressure, and protects against myocardial I/R injury in SHR. Am. J. Physiol.-Endocrinol. Metab..

[74] Machado Á.S., da Silva W., Souza M.A., Carpes F.P. (2018). Green tea extract preserves neuromuscular activation and muscle damage markers in athletes under cumulative fatigue. Front. Physiol..

[75] Lin D., Nussbaum M.A., Seol H., Singh N.B., Madigan M.L., Wojcik L.A. (2009). Acute effects of localized muscle fatigue on postural control and patterns of recovery during upright stance: Influence of fatigue location and age. Eur. J. Appl. Physiol..

[76] Jówko E., Długołęcka B., Makaruk B., Cieśliński I. (2015). The effect of green tea extract supplementation on exercise-induced oxidative stress parameters in male sprinters. Eur. J. Nutr..

[77] Wekesa A., Harrison M., Watson R. (2015). Physical activity and its mechanistic effects on prostate cancer. Prostate Cancer Prostatic Dis..

[78] Ichinose T., Nomura S., Someya Y., Akimoto S., Tachiyashiki K., Imaizumi K. (2011). Effect of endurance training supplemented with green tea extract on substrate metabolism during exercise in humans. Scand. J. Med. Sci. Sports.

[79] Carter S., Rennie C., Tarnopolsky M. (2001). Substrate utilization during endurance exercise in men and women after endurance training. Am. J. Physiol.-Endocrinol. Metab..

[80] De Bock K., Derave W., Eijnde B.O., Hesselink M., Koninckx E., Rose A.J., Schrauwen P., Bonen A., Richter E.A., Hespel P. (2008). Effect of training in the fasted state on metabolic responses during exercise with carbohydrate intake. J. Appl. Physiol..

[81] Holloszy J.O., Coyle E.F. (1984). Adaptations of skeletal muscle to endurance exercise and their metabolic consequences. J. Appl. Physiol..

[82] Murase T., Haramizu S., Shimotoyodome A., Tokimitsu I., Hase T. (2006). Green tea extract improves running endurance in mice by stimulating lipid utilization during exercise. Am. J. Physiol.-Regul. Integr. Comp. Physiol..

